# Seronegative primary Sjögren’s syndrome, a distinct subtype of primary Sjögren’s syndrome in Chinese patients

**DOI:** 10.1186/s41927-024-00384-9

**Published:** 2024-04-16

**Authors:** Jingying Lan, Chaoqiong Deng, Heqing Huang, Peishi Rao, Yangchun Chen, Yingying Shi, Jie Chen, Guixiu Shi, Yuan Liu, Shiju Chen

**Affiliations:** 1 Department of Rheumatology and Clinical Immunology, School of Medicine, The First Affiliated Hospital of Xiamen University, Xiamen University, Zhenhai Rd. 55#, 361000 Xiamen, Fujian China; 2 Xiamen Municipal Clinical Research Center for Immune Diseases, 361000 Xiamen, Fujian China; 3 Xiamen Key Laboratory of Rheumatology and Clinical Immunology, 361000 Xiamen, Fujian China; 4 Department of Rheumatology and Immunology, The First People’s Hospital of Yibin, 644000 Yibin, Sichuan China; 5 Department of Rheumatology and Clinical Immunology, Jiangxi Provincial People’s Hospital, The First Affiliated Hospital of Nanchang Medical College, 330006 Nanchang, China

**Keywords:** Primary Sjogren’s syndrome, Seronegative, Heterogeneity

## Abstract

**Background:**

To investigate the clinical and immune characteristics of patients with primary Sjögren’s syndrome (pSS) who were negative for anti–Sjögren’s-syndrome-related antigen A antibodies (anti-SSA) and anti–Sjögren’s-syndrome-related antigen B antibodies (anti-SSB) in Chinese population.

**Methods:**

A retrospective study were performed and 232 patients with pSS were analyzed. Patients positive for anti-SSA or/and anti-SSB were termed as seropositive pSS, and these negative for both anti-SSA and anti-SSB (non-antinuclear antibodies) as seronegative pSS. Clinical manifestations and laboratory findings were compared between the two groups.

**Results:**

Among the 232 patients with pSS, 192 (82.8%) were seropositive pSS and 40 (17.2%) were seronegative pSS. Compared to seropositive pSS, seronegative pSS were older and with higher percentage of low disease activity (ESSDAI < 5), xerostomia and xerophthalmia, with higher platelet count and level of creatine kinase. This subgroup was with lower levels of gamma globulin, immunoglobulin G, immunoglobulin A and autoantibodies including rheumatoid factor and antinuclear antibody in serum, and less immunoglobulin G deposition in labial gland.

**Conclusion:**

Seronegative pSS was a distinct subtype of pSS different from seropositive pSS. Clinical manifestations in seronegative pSS subgroup were restricted to exocrine gland and less B lymphocyte activation, while seropositive pSS were prone to present with systemic involvement and high disease activity. Specific underlying pathogenesis mechanisms and therapeutic strategies in this subgroup needed to be further studied.

## Introduction

Primary Sjögren’s syndrome (pSS) is a chronic systemic autoimmune disease characterized by lymphocytic infiltration and destruction of the exocrine glands including lacrimal and salivary glands, which results in sicca symptoms in affected patients [1, 2]. The clinical manifestations of this complex disease are heterogeneous and vary from xerostomia or xerophthalmia to systemic involvement [3, 4]. Though it has been aware that great heterogeneity exists in pSS patients diagnosed by the present criteria, little is known about the specific clinical characteristics of pSS subsets and the underlying mechanisms.

Anti-Sjögren’s-syndrome-related antigen A antibodies (anti-SSA; anti-Ro) and anti-Sjögren’s-syndrome-related antigen B antibodies (anti-SSB; anti-La) are characteristics biomarkers in pSS [5, 6]. However, current studies have demonstrated that approximately 15% of patients with pSS were negative for both anti-SSA and anti-SSB [7], who may be easily ignored by clinicians for pSS diagnosis. Although anti-SSA or anti-SSB play an important role in pSS pathogenesis [6, 8, 9], whether those pSS patients who are negative for anti-SSA and anti-SSB (non-antinuclear antibodies (ANA), which we termed seronegative pSS) share same pathogenesis mechanisms and clinical characteristics with those who are positive anti-SSA or anti-SSB (which we termed seropositive pSS) still needs to be explored.

In this study, we retrospectively analyzed specific clinical characteristics between seronegative pSS and seropositive pSS subgroups. The results demonstrated that seronegative pSS were distinct from seropositive pSS in clinical characteristics, indicating possible different underlying pathogenesis mechanisms. This may help a better understanding of heterogeneity in pSS.

## Patients and methods

### Study subjects

This retrospective study assessed the specific characteristics of patients with seronegative pSS. It was approved by the Clinical Research Ethics Committee of the First Affiliated Hospital of Xiamen University.

Patients diagnosed with pSS in the inpatient departments of Rheumatology in the First Affiliated Hospital of Xiamen University between January 2014 and July 2018 were reviewed. The only inclusion criterion was pSS and the diagnosis was based on 2002 American-European Consensus criteria [10]. A minor salivary gland was taken from the lower lip. Focal lymphocytic sialadenitis in a minor salivary gland biopsy with one or more foci of lymphocytes per 4 mm^2^ (focus score ≥ 1) was accepted as the histopathological criteria [11]. The exclusion criteria were as follows: combined head and neck radiation, chronic hepatitis C or human immunodeficiency virus infection, prior lymphoproliferative disorders, sarcoidosis, graft-versus-host disease, amyloidosis, IgG4-related disorders, and other systemic autoimmune diseases. A total of 232 patients with pSS were included in the study.

ANA were determined by immunofluorescence with a positive titer at 1/80 (Hep-2 ANA Test System; AESKU, Germany). Anti-CCP, anti-cardiolipin antibodies (anti-aCL) and anti-β2-GP1 were detected by ELISA (EUROIMMUN, Germany), and rheumatoid factor (RF) by immunoturbidity (Siemens, Germany). The autoantibody-targeted extractable nuclear antigens (ENAs) were detected by using immune blot method which identified 12 different target autoantigens including ds-DNA, U1RNP/Sm, Sm, SS-A, Ro52, SS-B, Scl-70, Jo-1, CENP-B, nucleosome, histones, ribosomal P-proteins (Rib) (EUROIMMUN, Germany). Anti-thyroglobulin antibody (anti-TGA), anti-thyroid peroxidase antibody (anti-TPO) and anti-thyrotrophin receptor antibody (anti-TRAb) were detected by chemiluminescence (Snibe, China). Anti-neutrophil cytoplasmic antibodies (anti-ANCA) were detected by immunofluorescence and the targeted autoantigens by immunoblotting (EUROIMMUN, Germany).

### Data collection

Information about patients’ histories, laboratory findings, images, histological findings and treatment regimen were obtained from the electronic medical records of patients. Laboratory findings including routine blood examinations, routine urine assessments, erythrocyte sedimentation rate (ESR), C-reactive protein (CRP) and conventional hepatorenal function examinations were collected. Laboratory parameters related to immunological characteristics such as complement 3 (C3), complement 4 (C4), gamma globulins and autoantibodies were also collected. In addition, disease activity of pSS in all patients were assessed by the European League Against Rheumatism Sjögren’s Syndrome Disease Activity Index (ESSDAI) [12]. Complications including primary biliary cirrhosis, autoimmune thyroid disease, immune thrombocytopenia (ITP) and interstitial lung disease were also noted.

### Statistical analysis

All statistical analysis were performed by using SPSS software. The S-W (Shapiro-Wilk) test was used to detect normal distribution. Data were shown as median with 25th–75th percentiles (Q25–Q75) based on the means of distribution. Categorical data were expressed as positive number/test number (%). For comparisons between two groups, the chi-squared or Fisher’s exact tests were used for binary data and the Student’s t- or Mann-Whitney U tests were used for continuous data. *P* < 0.05 were considered statistically significant.

## Results

### Basic characteristics of total pSS patients included in the present study

In total, 232 patients with pSS were retrospectively analyzed with mean age of 50.00 (40.25-60.00). Among these patients, 82.80% (192/232) were seropositive and 40 (17.2%) were seronegative. Anti-SSA was presented in 81.5% (189/232) patients and anti-SSB in 32.3% (75/232) patients. Other autoantibodies including ANA and RF were presented in 88.4% (205/232) and 24.7% (54/219) of patients with pSS, respectively.

### Clinical characteristics of seronegative pSS compared to seropositive pSS

Clinical characteristics of seronegative pSS were shown in Table 1. The diagnosis age of seronegative pSS was 56.5 (50.00-63.75), significantly older than patients with seropositive pSS (*p* = 0.000). Compared to seropositive pSS, the presence of symptoms related to glandular dysfunction including xerostomia (80.00% vs. 57.80%, *p* = 0.009) and xerophthalmia (62.50% vs. 44.80%, *p* = 0.041) were more common in seronegative pSS (Table 1). The prevalence of hematological involvement including ITP and leukopenia were higher in seropositive pSS, though the difference was not significant (*p* > 0.05). No obvious differences were observed in interstitial lung disease (ILD) between two pSS subgroups (42.50% vs. 40.40%, *p* = 0.860). However, pattern of ILD appeared to be different among patients with seropositive and seronegative pSS. The percentage of non-specific interstitial pneumonia (NSIP) was higher in seropositive pSS (87.84% vs. 68.75%, *p* = 0.056), and the percentage of usual interstitial pneumonia (UIP) was lower in this group (1.35% vs. 12.50%, *p* = 0.080), though the differences were not statistically significant. There were no statistically differences in other complications including pulmonary arterial hypertension, peripheral or central neuropathy, arthropathy, renal interstitial diseases and hypokalemia. The percentage of low disease activity (ESSDAI score < 5) was significantly higher in seronegative pSS (*p* = 0.013).

**Table 1:** Comparison of clinical characteristics of patients with pSS

	Seronegative pSS (*n* = 40)	Seropositive pSS (*n* = 192)	***P***-value
**Basic characteristic**			
Age at diagnosis (years)	56.5 (50.00-63.75)	48.00 (39.00–58.00)	0.000^*^
Gender (female/male)	36/4	176/16	0.974
Course of disease (months)	36 (12.00-117.00)	24 (5.00–60.00)	0.042^*^
**Clinical manifestations**			
Xerostomia	32/40 (80.00)	111/192 (57.80)	0.009^*^
Xerophthalmia	25/40 (62.50)	86/192 (44.80)	0.041^*^
Schirmer test positivity	24/26 (92.30)	85/110 (77.30)	0.084
Saprodontia	7/40 (17.50)	19/192 (9.90)	0.266
Cutaneous manifestations	3/40 (7.50)	28/192 (14.60)	0.231
Interstitial lung disease	16/40 (42.50)	74/183 (40.40)	0.860
NSIP	11/16 (68.75)	65/74 (87.84)	0.056
OP	3/16 (18.75)	8/74 (10.81)	0.405
UIP	2/16 (12.50)	1/74 (1.35)	0.080
Pulmonary arterial hypertension	1/19 (5.30)	3/74 (4.10)	1.000
ITP	2/40 (5.00)	28/192 (14.60)	0.100
Anemia	13/40 (32.50)	66/192 (34.40)	0.820
Leukopenia	4/40 (10.00)	27/192 (14.10)	0.492
Peripheral neuropathy	1/40 (2.50)	9/192 (4.70)	0.848
Central neuropathy	1/40 (2.50)	6/192 (3.10)	1.000
Arthropathy	11/40 (27.50)	35/192 (18.20)	0.181
Renal interstitial disease	0/40 (0.00)	4/192 (2.10)	1.000
Hypokalemia	2/40 (5.00)	30/192 (15.60)	0.076
Primary biliary cirrhosis	2/40 (5.00)	4/192 (2.10)	0.276
Infection	12/40 (30.00)	51/192 (26.60)	0.657
ESSDAI score < 5	13/40 (32.50)	30/192 (15.63)	0.013^*^
**Laboratory findings**			
Hemoglobin, g/L	125.50 (110.25-136.75)	123.00 (111.00-132.00)	0.268
Leukocyte, X10^9^/L	6.32 (4.70–7.91)	5.50 (4.14–7.28)	0.258
Platelet, X10^9^/L	265.0 (205.25-310.75)	218.5 (162.50-272.75)	0.003^*^
ESR, mm/h	16.50 (11.00–54.00)	33.00 (17.00–54.00)	0.044^*^
CRP, mg/dL	1.00 (0.54–5.75)	1.90 (0.61–5.80)	0.460
ALT, U/L	20.00 (14.0–32.0)	18.00 (13.00–28.00)	0.230
AST, U/L	20.00 (15.00-32.50)	19.00 (15.00–27.00)	0.873
Serum total bilirubin, U/L	10.85 (7.48–15.40)	8.00 (5.90–10.90)	0.005^*^
FT3, U/L	4.40 (3.61–4.65)	4.27 (3.70–4.70)	0.955
FT4, U/L	14.28 (12.56–15.60)	14.67 (12.68–16.15)	0.521
TSH, U/L	2.09 (1.41–3.27)	2.13 (1.27–3.50)	0.968
Creatine kinase, U/L	62.00 (33.75-103.25)	48.00 (30.00-67.75)	0.026^*^
Creatinine, umol/L	49.5 (42.25-61.00)	54.0 (45.0-61.25)	0.179
Immunosuppressive drugs			
Glucocorticoid	23/40(57.50)	156/192(81.30)	0.001^*^
DMARDs	35/40(87.50)	184/192(95.80)	0.037^*^
Comorbidities	10/40 (25.00)	73/192 (38.00)	0.118

Continuous data were presented as median with 25–75th percentiles and categorical data as positive number/test number (%). ESSDAI, European League Against Rheumatism Sjögren’s Syndrome Disease Activity Index; ITP, immune thrombocytopenia; RTA, renal tubular acidosis; ESR, erythrocyte sedimentation rate; CRP, C-reactive protein. DMARDs, disease modifying antirheumatic drugs which included hydroxychloroquine, methotrexate, cyclophosphamide, mycophenolate mofetil, cyclosporine, and leflunomide; Comorbidities including gallstone, hypertension, diabetes, chronic HBV infection, old pulmonary tuberculosis, hyperlipidemia and fatty liver; *, *P* value < 0.05

Laboratory findings of seronegative pSS and seropositive pSS patients were also compared (Table 1**)**. Level of ESR was lower in seronegative pSS (*p* = 0.044), while parameters including platelet count (*p* = 0.003), serum total bilirubin (*p* = 0.005), creatine kinase (*p* = 0.026) were higher in seronegative pSS. No significant differences were showed between seronegative pSS and seropositive pSS in other laboratory parameters such as hemoglobin, leukocyte count in periphery blood, level of creatinine, CRP, and variables related to liver function, renal function and thyroid function.

### Immunological features of patients with seronegative pSS

A comparison of immunological features between seronegative and seropositive pSS was shown in Table 2. Parameters related to B lymphocyte activation including level of gamma globulin (*p* = 0.012), immunoglobulin G (*p* = 0.000) as well as immunoglobulin A (*p* = 0.027) were significantly lower in seronegative pSS. Though it seemed that the level of IgM was higher in seronegative pSS, the difference did not reach the statistical significance. Presence of ANA (*p* = 0.000) and RF (*p* = 0.017) were significantly lower in seronegative pSS, while no significant differences were detected in other autoantibodies such as anti-CCP, anti-dsDNA, anti-histones, anti-nucleosome, anti-U1nRNP/Sm, anti-Rib, anti-Sm, anti-Jo1, anti-CENPB, anti-ANCA, anti-aCL and anti-β2-GP1 (partial data not shown in Table 2).

**Table 2:** Comparison of immunological characteristics of patients with pSS

	Seronegative pSS (*n* = 40)	Seropositive pSS (*n* = 192)	***P***-value
Low Complement 3	10/40 (25.00)	66/192 (34.40)	0.250
Low Complement 4	0/40 (0.00)	17/192 (8.90)	0.105
Gamma globulin, g/L	30.10 (26.50–35.00)	32.80 (28.95–37.95)	0.012^*^
Immunoglobulin A, g/L	2.47 (1.87–3.50)	2.98 (2.26–3.93)	0.027^*^
Immunoglobulin G, g/L	14.20 (12.20–15.70)	17.45 (13.83–21.45)	0.000^*^
Immunoglobulin M, g/L	1.21 (0.89–2.07)	1.17 (0.81–1.56)	0.172
ANA positivity	22/40 (55.00)	183/192 (95.30)	0.000^*^
RF positivity	4/40 (10.00)	50/179 (27.90)	0.017^*^
Anti-CCP positive	1/30 (3.30)	7/138 (5.10)	1.000
Anti-TGA positive	4/13 (30.80)	16/86 (18.60)	0.517
Anti-TPO positivity	8/13 (61.50)	26/84 (31.00)	0.066
Anti-TRAb positivity	0/7 (0.00)	5/52 (9.60)	1.000

Continuous data were presented as median with 25–75th percentiles and categorical data as positive number/test number (%). ANA, anti-nuclear antibody positivity; RF, Rheumatoid factor; CCP, cyclic citrullinated peptides antibody; anti-TGA, anti-thyroglobulin antibody; anti-TPO, anti-thyroid peroxidase antibody; TRAb, thyrotrophin receptor antibody. *, *P* value < 0.05

### Histological characteristics of labial gland in patients with seronegative pSS

Patients with seronegative pSS were all positive for lymphocytic infiltration in labial gland biopsy, which was more common than that of seropositive pSS (100.00% vs. 79.80%, *p* = 0.002). Among patients with available immunohistochemical information of labial glands, histological characteristics of labial gland in seronegative pSS were also analyzed (Table 3). IgG deposition in labial gland was more common in seropositive pSS compared with seronegative pSS (*p* = 0.014). No obvious differences were found in infiltration of T and B lymphocytes between two groups.

**Table 3:** Comparison of histological characteristics of patients with pSS

	Grading	Seronegative pSS	Seropositive pSS	***P***-value
		(*n* = 21)	(*n* = 48)	
CD4	+	8(38.09)	9(19.15)	0.257
	++	3(14.29)	10(21.28)	
	+++	10(47.62)	28(59.58)	
CD8	+	13(61.90)	23(47.91)	0.233
	++	0(0.00)	3(6.25)	
	+++	8(38.10)	22(45.83)	
CD38	+	10(52.63)	17(36.17)	0.328
	++	1(5.26)	1(2.13)	
	+++	8(42.11)	29(61.70)	
IgG	+	16(84.21)	22(47.83)	0.014^*^
	++	0(0.00)	2(4.35)	
	+++	3(15.79)	22(47.83)	
CD20	+	5(38.46)	6(26.09)	0.681
	++	2(15.38)	3(13.04)	
	+++	6(46.16)	14(60.87)	

## Discussion

Patients with pSS are highly heterogenous. Anti-SSA and anti-SSB are most widely used immunological biomarkers in pSS diagnosis, but some of patients with pSS are negative for both autoantibodies. Limited studies analyzed the clinical characteristics of pSS seronegative subgroup. It has been proposed in 2017 that there is an urgent need to define “seronegative pSS” [13]. Our present study explored clinical heterogeneity of Chinese pSS patients and found seronegative pSS were significantly different from seropositive pSS in several aspects which may indicate possible distinct underlying pathogenetic mechanisms.

Compared with seropositive pSS, we found that Chinese patients with seronegative pSS were significantly older, with clinical manifestations mainly restricted to exocrine gland dysfunction such as xerostomia and xerophthalmia, while lower presence of systemic involvement and more presence of low disease activity (ESSDAI < 5). Similar to the present study, previous studies also explored the heterogeneity of patients with pSS in the perspective of SSA/SSB, as summarized in Table 4. Though there were some differences in defining seronegative subgroup among studies, consistent distinct clinical characteristics of seronegative pSS were unraveled by those studies [7, 14–19]. In general, patients with seronegative pSS were significantly older at diagnosis, with symptoms more specific to exocrine gland, with lower presence of systemic involvement and lower risk of lymphoma, while patients with seropositive pSS were younger at diagnosis, with higher presence of systemic involvement such as leucopenia, higher level of immune parameters indicating B lymphocyte activation such as hypergammaglobulinemia.

**Table 4:** Summary of the results of seronegative and seropositive pSS in published studies

Author	Year	Country	Subgroup (n)	Study type	Result
Veli Yazisiz [7]	2021	Turkey	**seropositive group (317)**: Anti-Ro+/Anti-La+/ANA+/RF+. **seronegative group (58)**: negative for all four autoantibodies	Retrospective	No statistically significant differences in terms of patient age, age at diagnosis, sex distribution, clinical features, and laboratory findings were found between seronegative and seropositive pSS. The frequency of hypergammaglobulinemia was higher in seropositive pSS.
G. Cafaro [19]	2020	Italy	Patients with anti-Ro and positive SGB were included. **SSB- group (319)** **SSB + group (281)**	Retrospective	Anti-SSB positive patients were younger at disease diagnosis and had a longer disease duration, had a higher prevalence of hypergammaglobulinaemia and circulating rheumatoid factor and of lymphoproliferative disorders in comparison to seronegative group.
Jowy Tani [14]	2020	Taiwan, China	**Seronegative group** [10]: SSA- and SSB- **seropositive group(28)**: SSA + or SSB+	Retrospective	Thermal QST showed more prominent abnormalities in seronegative pSS compared to seropositive pSS, while seronegative pSS showed much less prominent motor axonal changes and no significant sensory axonal changes.
Y. Park [17]	2019	Korea	**SSA + group (326)**, **SSA- group (29)**	Retrospective	The anti-SSA negative group showed less rheumatoid factor positivity, leucopenia, hypergammaglobulinemia, lower serum β2-microglobulin level, more anti-centromere antibody positivity, higher score in dryness domain of EULAR SS patient-reported index and more positivity for peripheral nervous system domain in EULAR SS disease activity index and loss of teeth than patients who were anti-Ro/SSA positive.
Ewa Kontny [18]	2018	Poland	**Group 1** [15]: SSA- and SSB- **Group 2 (27)**: SSA + only **Group 3 (39)**: SSA + and SSB+	Retrospective	Patients of groups 2 and 3 developed disease symptoms at younger age, and more often had positive Schirmer’s test and skin lesions, higher frequency of autoantibodies other than anti-SSA and anti-SSB, higher serum concentrations of APRIL than those of group 1.
Luca Quartuccio [16]	2015	Italy	**seronegative group (206)**: SSA-and SSB- **seropositive group (342)**: SSA + and/or SSB+	Retrospective	Variables statistically associated with seropositive pSS were younger age at diagnosis, glandular swelling, purpura, leucopoenia, lymphoma, low C3, low C4, hypergammaglobulinemia, ANA, rheumatoid factor, and serum cryoglobulins. Seronegative pSS appears to be characterized by a lower risk of lymphoma and by a lower level of B-cell expansion.
Barbara M. Segal [15]	2013	America	**Seropositive group (68)**: SSA + or SSB+. **Seronegative group (40)**: SSA- and SSB-.	Retrospective	Chronic pain, defined as daily pain for > 3 months, was reported by 65% of seropositive and 75% of seronegative patients. Pain severity was greater and physical function was reduced in the seronegative patients. Prevalence of neuropathic pain, depression, anxiety, and disability was similar between groups.

Anti-SSA and anti-SSB antibodies were presented in up to 75% of patients with pSS [6]. The American–European Consensus Group criteria and the ACR criteria recognized the histopathology and the presence of the anti-SSA and/or anti-SSB as the cornerstones for classification of pSS [5, 20, 21]. Previous studies reported that the anti-SSA antibody was related to disease-specific symptoms and disease severity in pSS [6, 22]. Anti-SSB, as another common autoantibody in patients with pSS, seldomly existed alone without anti-SSA in pSS [6, 23]. The present study and previous studies demonstrated that seronegative pSS were clinically distinct from patients with seropositive pSS, indicating different underlying pathogenesis mechanisms.

A recent study found that patients with seronegative pSS and seropositive pSS were quite different in genetic features. Several variants in the HLA region were found to be unique to patients with seropositive pSS [24]. Some observations on epigenetic regulation have also reported the upregulated expression of IFN-induced genes, for example, the IFN signature, was mainly seen in patients with seropositive pSS [24–26]. Those studies further hinted that seronegative pSS was a distinct subset of pSS from seropositive pSS in pathogenesis mechanisms, which may need to be investigated separately in clinical trials or prognostic study.

There were several limitations in our study. Firstly, this study was performed in single center in China and the sample size included was relatively small, especially the seronegative pSS. This might underestimate the role of anti-SSA or anti-SSB in pathogenesis of some systemic involvement such as lung involvement, as our data showed that presence of NSIP was numerically higher in seropositive pSS but the difference was not significant. Secondly, limited by retrospective nature of this study, our results could have been compromised by the bias due to the missing of certain clinical information such as lung function. Thirdly, clinical characteristics of seronegative pSS may change over time and patients was not followed-up, the different outcomes including infection, malignancy, response to immunosuppressive treatment were unclear. More multi-center prospective studies are needed in future to validate our results and clarify the clinical features of this distinct subtype of pSS.

In conclusion, seronegative pSS was a distinct subtype of pSS different from seropositive pSS. Clinical manifestations of patients with seronegative pSS were restricted to exocrine gland, while seropositive pSS were prone to present with systemic involvement and high disease activity. Considering these features, different therapeutic strategies and potential underlying different pathogenesis mechanisms in seronegative pSS need to be further investigated.

## Data Availability

Data are available upon reasonable request.
